# “Me Cuido Activo Manizales”: A Community Care Model Based on an Active Aging Framework for Elderly People in Colombia

**DOI:** 10.1093/geroni/igaa057.304

**Published:** 2020-12-16

**Authors:** Lina Gonzalez Ballesteros, Santiago López Zuluaga, Ana María Ortiz Hoyos, Oscar Gómez, Lina GonzalezBallesteros

**Affiliations:** 1 Physician, Bogotá, Colombia; 2 Sociologist, Bogotá, Colombia; 3 Psicologist, Bogotá, Distrito Capital de Bogota, Colombia; 4 Physician, Bogotá, Distrito Capital de Bogota, Colombia

## Abstract

Colombia, as other countries in the Latin American region has aged at a higher rate than countries from developed ones. This challenges the way these countries decide how to attend the ageing of their population. “Me cuido activo Manizales” is a community care model based on an active ageing framework developed in Manizales, Colombia in 2017. It is based on collaborative and experiential learning activities for elderly people and their caregivers in the areas of care and self-care, writing and memory, healthy habits, physical activity, resilience and rights, and economic productivity. A before and after analysis of the 88% of enrolled subjects that participated through 2017-2019 (Median age = 70; IQR = 76 years) show stable results in independency (Barthel before = 97,23 Vs Barthel after = 94,68; t = 1,48; p = 0,14), instrumental activities of daily living (Lawton-Brody before = 3,63 Vs Lawton-Brody after = 3,58; t = 0,748; p = 0,47), resilience (CD-RISC10 before = 28,61 Vs CD-RISC10 after = 28,28; t = 0,40; p = 0,69) and MMSE scores (before = 25,72 Vs after = 24,62; t = 1,67; p = 0,10). Qualitative analysis evidenced increased awareness of self-care lifestyles and active ageing, and the need for institutional presence providing logistic and personnel support and better ways to engage groups rather than the individual. We believe this is a holistic approach that focuses on key aspects of the current perception of elderly functionality and that it engages individuals and communities along in a self-aware healthy ageing.

